# Functional Characterization of the Vitamin K_2_ Biosynthetic Enzyme UBIAD1

**DOI:** 10.1371/journal.pone.0125737

**Published:** 2015-04-15

**Authors:** Yoshihisa Hirota, Kimie Nakagawa, Natsumi Sawada, Naoko Okuda, Yoshitomo Suhara, Yuri Uchino, Takashi Kimoto, Nobuaki Funahashi, Maya Kamao, Naoko Tsugawa, Toshio Okano

**Affiliations:** 1 Department of Hygienic Sciences, Kobe Pharmaceutical University, Kobe, Japan; 2 Faculty of Pharmaceutical Sciences, Suzuka University of Medical Science, Suzuka, Japan; 3 Department of Bioscience and Engineering, Shibaura Institute of Technology, Saitama, Japan; Nihon University School of Medicine, JAPAN

## Abstract

UbiA prenyltransferase domain-containing protein 1 (UBIAD1) plays a significant role in vitamin K_2_ (MK-4) synthesis. We investigated the enzymological properties of UBIAD1 using microsomal fractions from Sf9 cells expressing UBIAD1 by analysing MK-4 biosynthetic activity. With regard to UBIAD1 enzyme reaction conditions, highest MK-4 synthetic activity was demonstrated under basic conditions at a pH between 8.5 and 9.0, with a DTT ≥0.1 mM. In addition, we found that geranyl pyrophosphate and farnesyl pyrophosphate were also recognized as a side-chain source and served as a substrate for prenylation. Furthermore, lipophilic statins were found to directly inhibit the enzymatic activity of UBIAD1. We analysed the aminoacid sequences homologies across the menA and UbiA families to identify conserved structural features of UBIAD1 proteins and focused on four highly conserved domains. We prepared protein mutants deficient in the four conserved domains to evaluate enzyme activity. Because no enzyme activity was detected in the mutants deficient in the UBIAD1 conserved domains, these four domains were considered to play an essential role in enzymatic activity. We also measured enzyme activities using point mutants of the highly conserved aminoacids in these domains to elucidate their respective functions. We found that the conserved domain I is a substrate recognition site that undergoes a structural change after substrate binding. The conserved domain II is a redox domain site containing a CxxC motif. The conserved domain III is a hinge region important as a catalytic site for the UBIAD1 enzyme. The conserved domain IV is a binding site for Mg^2+^/isoprenyl side-chain. In this study, we provide a molecular mapping of the enzymological properties of UBIAD1.

## Introduction

Natural vitamin K has two molecular homologues: plant-derived vitamin K_1_ (phylloquinone: PK), which contains a phytyl group side chain, and bacterial-derived vitamin K_2_ (menaquinone-n: MK-n), which contains a polyisoprenyl side chain. Menadione (MD) is a synthetic compound that lacks a side chain. All forms of vitamin K share a common 2-methyl-1,4-naphthoquinone nucleus. Vitamin K is an essential cofactor required for γ-glutamyl carboxylase that converts specific glutamic acid residues into γ-carboxyglutamic acid residues in proteins involved in blood-clotting and bone metabolism [1, 2]. In addition, vitamin K is required for the synthesis of other calcium-binding proteins such as the bone Gla protein (osteocalcin), matrix Gla-protein, protein S and the growth arrest specific gene 6 proteins [3–5]. Besides a role as a cofactor for γ-glutamyl carboxylase, vitamin K is involved in the transcriptional regulation of the nuclear receptor SXR/PXR [6–8] and regulates PKA signalling [9]. Vitamin K functions as a mitochondrial electron carrier during ATP production in the electron transport chain [10, 11].

One of the major forms of vitamin K in humans, MK-4, is produced by cleavage of the phytyl side chain from dietary PK to release MD in the intestine, followed by delivery of MD through the mesenteric lymphatic system and blood circulation to tissues where it is then converted to MK-4 by a prenyltransferase such as UbiA prenyltransferase domain-containing protein 1 (UBIAD1) with geranylgeranyl diphosphate (GGPP) [12–14]. Recently, it has been reported that UBIAD1 catalyses the non-mitochondrial synthesis of coenzyme Q10 (CoQ10) in zebrafish [15]. CoQ10 exists in several forms and can be found in microorganisms, plants and mammals. CoQ9 is largely found in rats and mice, whereas CoQ10 is prevalent in humans and zebrafish. CoQ10 is an endogenously synthesized electron carrier that is critical for electron transfer in the mitochondrial membrane for respiratory chain activity, and as a lipid-soluble antioxidant, it plays an important role in protecting biological membranes from oxidative damage.

UBIAD1 exhibits various subcellular localisations, including the endothelial reticulum [14, 15], Golgi complex [15, 16] and mitochondria [17], in a variety of tissues and cell types of vertebrates. Whether UBIAD1 has other functions beside the synthesis of MK-4 is unknown. *UBIAD1/ubiad1* mutations in zebrafish have been reported to cause cardiac oedema and cranial haemorrhages [16, 18] and *UBIAD1/heixuedian* mutations in *Drosophila* cause defects in mitochondrial ATP production [10, 19]. Missense mutations in *UBIAD1* are the underlying cause of the human genetic disorder Schnyder corneal dystrophy (SCD). SCD causes abnormal deposition of cholesterol and phospholipids in the cornea, eventually leading to blindness [20]. UBIAD1, also known as transitional epithelial response protein 1 (TERE1), suppresses the proliferation of transitional cell carcinoma cell lines and prostate cancer cell lines [21–25].

UBIAD1 belongs to the membrane prenyltransferase family. Membrane prenyltransferases are involved in the synthesis of ubiquinones [26], menaquinones [27], chlorophylls [28], archaeal lipids [29], numerous prenylated plant flavonoids [30, 31] and vitamin E [32, 33]. Membrane prenyltransferases catalyse the substitution of the prenyl acceptor, leading to the formation of benzo-quinones and naphtho-quinones as well as prenylated polyphenols, which play various important biological roles in organisms ranging from bacteria to humans. Membrane prenyltransferases have two characteristic conserved motifs with the consensus sequences NDxxDxxxD and DxxxD, often referred to as the first and second aspartate-rich motifs, respectively.

Recently, Cheng *et al*. and Huang *et al*. have defined the crystal structure of UbiA derived from archaebacteria, followed by the detailed structural analysis of the UbiA homolog from *Aeropyrum pernix* and UbiA homolog from the extremophile *Archaeoglobus fulgidus* (AfUbiA) [34, 35]. However, no detailed information regarding the prenylation domain and substrate recognition site in the aminoacid sequence of human UBIAD1 is available. Therefore, the present study utilized UBIAD1 to characterize the enzymatic function of this family of proteins by site-directed mutagenesis, because UBIAD1 has several advantages over other membrane prenyltransferase. For example, the missense mutation of UBIAD1 responsible for the human genetic disorder SCD was used to investigate changes in enzyme activities and enabled the identification of a key structural site for the MK-4 synthetic activity of UBIAD1. Based on this initial knowledge, we performed an aminoacid sequence homology search across the menA and UbiA families to identify structural patterns in the UBIAD1 protein and to focus on the highest conserved domains and aminoacids. The conservation of residues mutated, which were changed for MK-4 synthetic activity. In this article, we describe the importance of different structural domains in UBIAD1 and their functional correlation with MK-4 biosynthetic activity.

## Materials and Methods

### Materials

D-labelled MD (MD-d_8_) was purchased from C/D/N Isotopes, Inc. (Quebec, Canada). PK-d_7_, MK-4-d_7_, MK-3-d_7_, MK-2-d_7_, MK-4 epoxide and ^18^O-labelled MK-4 (MK-4-^18^O) were respectively synthesized in our laboratory as reported previously [13]. Geranyl diphosphate (GPP), farnesyl pyrophosphate (FPP) and GGPP were purchased from Sigma Chemical Co., (St Louis, MO, USA). Lovastatin (LOV), simvastatin (SIM), pravastatin sodium salt (pravastatin: PRA) and etidronate disodium (etidronate: ETI) were purchased from Wako Pure Chemical Industries, Ltd. (Osaka, Japan). Alendronate monosodium trihydrate (alendronate: ALN) and risedronate sodium (risedronate: RIS) were purchased from LKT Laboratories (St. Paul, MN, USA). Zoledronate disodium salt and tetrahydrate (zoledronate: ZOL) were purchased from Toronto Research Chemicals (Toronto, ON, Canada). ^13^C-labelled *para*-hydroxybenzoate (^13^C_6_-PHB) was purchased from Santa Cruz Biotechnology (Santa Cruz, CA, USA). CoQ9, CoQ10, solanesol and decaprenol were donated by Eisai Co., Ltd. (Tokyo, Japan). Organic solvents of HPLC grade were purchased from Nacalai Tesque (Kyoto, Japan). Other chemicals were used of the highest quality commercially available.

### Ethics statement

All cell experimental protocols were performed in accordance with the Guidelines for Cell Experiments at Kobe Pharmaceutical University and were approved by The Ethics Committee of Kobe Pharmaceutical University, Kobe Japan.

### Cell Culture

*Spodoptera frugiperda* (Sf9) cells were purchased from Invitrogen (Carlsbad, CA, USA) [14]. Sf9 cells were grown at 28°C in a tissue culture flask in Grace’s insect medium (Invitrogen) supplemented with 10% FCS (Gibco/BRL Life Technologies, Grand Island, NY, USA). Human osteosarcoma MG63 cells were provided by K. Ozono [14]. MG63 cells were maintained in Dulbecco’s modified Eagle’s medium (Nakalai Tesque) supplemented with 1% penicillin, 1% streptomycin and 10% FCS (Gibco/BRL) in a carbon dioxide (CO_2_) incubator at 37°C (5% CO_2_).

### UBIAD1 and UBIAD1 mutant expression in Sf9 cells

The cDNA for the UBIAD1 coding region was cloned into pIEx/Bac-1 (Merck & Co., Whitehouse Station, NJ, USA) in Sf9 cells using Insect GeneJuice (Merck). UBIAD1 mutant variant were generated by site-directed mutagenesis, which was performed using the QuikChange site-directed mutagenesis kit (Stratagene, La Jolla, CA, USA) according to the manufacturer’s instructions. The QuikChange primers are listed in S1 Table. A recombinant baculovirus expressing UBIAD1 and the UBIAD1 mutant was generated using the BacMagic system (Merck) according to the manufacturer’s protocol. To achieve the expression of UBIAD1 (wild type) and UBIAD1 mutants, cells (2 × 10^6^ cells/ mL) were infected of recombinant baculovirus, followed by a 72 h expression period. Double point mutants were generated using the same method.

### Preparation of microsomal fraction

All the steps in the preparation of the microsomal membrane fraction were performed at 4°C. Sf9 cells were thawed and resuspended in 10× cell volume of sonication buffer containing 100 mM Tris-HCl (pH 7.4), 500 mM NaCl and 1% proteinase inhibitor cocktail. The cell suspension was sonicated for 5 min and centrifuged at 3,000 × g for 10 min to remove unbroken cells and nuclei. The supernatant was centrifuged at 105,000 × g for 60 min. The microsome pellet was resuspended in ice-cold buffer A containing 100 mM Tris-HCl (pH 8.5) and protease inhibitor cocktail (Roche, Lewes, UK). The microsomal membrane suspension was homogenized and stored at −80°C.

### SDS/PAGE and immunoblotting analysis

SDS/PAGE and immunoblotting of microsomal membrane proteins were performed according to a method reported previously [14], with slight modifications. In brief, proteins were subjected to SDS/ PAGE (12.5% gel) and then transferred onto Hybond-PVDF membrane (GE Healthcare, Hatfield, UK). For blocking, the membrane was treated with Blocking One (Nacalai Tesque) overnight at room temperature. Then, the membrane was incubated with rabbit anti-UBIAD1 polyclonal antibody against an UBIAD1-specific peptide (CPEQDRLPQRSWRQK-COOH) (MRL Co., Ltd. Woburn, MA, USA). Subsequently, a donkey peroxidase-conjugated secondary antibody was used (SantaCruz) and the UBIAD1 protein was detected using an electro chemiluminescent detection system (BioRad Laboratories, Hercules, CA, USA).

### Assay for prenyltransferase activity

Reactions were performed in 1 mL of 100 mM Tris-HCl (pH 8.5) containing 1 mM DTT, 100 nM GGPP, 100 nM MD-*d*_8_ and 5 μg protein of Sf9-UBIAD1 microsomes. Reaction mixtures were incubated at 37°C for 3 h, and the reactions were stopped by the addition of 1 mL of ethanol. Metabolites were extracted with 3 mL of hexane and 1 mL of ethanol containing the internal standard, and the metabolites were analysed by LC-APCI-MS/MS as previously described [12–14]. The optimum pH was obtained using PIPES-buffer (pH 6.0–7.5), Tris-buffer (pH 7.0–9.5) or CHES-buffer (pH 9.0–10.5). DTT requirement was evaluated at 0, 0.033, 0.067, 0.1, 0.2, 0.5, and 1 mM concentrations. Side-chain selectivity for Mg^2+^ was examined by adding MgCl_2_ at 0, 0.1, 1, 10, and 30 mM concentrations. We also examined the inhibitory effect of statins and bisphosphonates on UBIAD1enzyme activity by adding statins at 0.5, 1, 5 or 10 × 10^−6^ M and bisphosphonates at 5, 10, 50 or 100 × 10^−6^ M. The amount of MK-4-d_7_ converted from MD-d_8_ per expression amount of the UBIAD1 protein was calculated and then divided by the reaction time. The obtained value was defined as ‘UBIAD1 activity’.

### Effects of statins and bisphosphonates on MK-4 synthesis in MG63 cells

MG63 cells were cultured for 2 days on six-well tissue culture plates (4 × 10^5^ cells/well) and treated for 24 h with the culture medium containing 0.5, 1, 5 or 10 × 10^−6^ M statins or 5, 10, 50 or 100 × 10^−6^ M bisphosphonates. Subsequently, the cells were supplemented with 1 mM MD-d_8_ with or without 1, 2 or 5 × 10^−6^ M GGPP at 37°C for 24 h. Cells were collected and washed three times with cold PBS (free of Ca^2+^ and Mg^2+^) and then stored at −30°C. After a heating step to reach 20–25°C, cells were lysed in 1 ml of PBS(−). Cell lysates (20 μL) were analysed for protein concentrations. ^18^O-labelled PK and ^18^O-labelled MK-4 were added as internal standards to the cell lysates in brown screw-capped tubes. MK-4-d_7_ and MK-4-d_7_ epoxide were measured by the LC-APCI-MS/MS method described above.

### Analysis of aminoacid sequences homologies across menA and UbiA families

We analysed the homology of the aminoacid sequences across the menA and UbiA families. The menA family consists of representative UBIAD1s of several species, including human (NP_037451.1), chimpanzee (JAA33188.1), horse (XP_001492378), mouse (NP_082149.1), rat (NP_001101463.1), dog (XP_544571.1), bovine (XP_002694102.1), bird (NP_001026050.1), zebrafish (NP_001186655.1), drosophila (AAL34085.1) and *Escherichia coli* menA (YP_491521.1). The UbiA family includes the human ubiquinone biosynthetic enzyme COQ2 (AAH08804.1), yeast ubiquinone biosynthetic enzyme coq2 (CAA96321.1), *E*. *coli* ubiquinone biosynthetic enzyme ubiA (YP_492183.1) and *Lithospermum erythrorhizon* PHB geranyltransferase LePGT1 (Q8W405). Alignments were performed using the ClustalW program in KEGG (http://www.genome.jp/tools/clustalw/), and the phylogenetic tree was constructed using Tree View (http://taxonomy.zoology.gla.ac.uk/rod/treeview.html). The homology analysis with ClustalW was followed by a search of highly homologous aminoacids using GeneDoc (http://www.nrbsc.org/gfx/genedoc/).

### Enzyme kinetics analysis

For the enzyme kinetics analysis of MD-d_8_, 100 nM of MD-d_8_ was added in the presence of 5, 10, 20, 50 or 100 nM GGPP. The Michaelis–Menten equation was calculated with the MK-4-d_7_ production levels, and then a Lineweaver–Burk plot was constructed. GPP, FPP and GGPP were each added at a concentration of 5, 10, 20, 50 or 100 nM in the presence of 100 nM of MD-d_8_. The Michaelis–Menten equation was calculated with MK-2-d_7_, MK-3-d_7_ or MK-4-d_7_ production levels, and then the respective Lineweaver–Burk plots were constructed. Based on this, we determined the maximum rate (*V*max) and the Michaelis constant (*K*m) of the enzyme.

### Construction of stable UBIAD1 or UBIAD1 mutant expression vectors for MG63 cells

A pEBMulti-neo vector (Wako) was used for the stable expression of UBIAD1 or its mutant variants in MG63 cells. The PCR templates used for the construction of stable expression vectors were pIEx/Bac-1-UBIAD1 and pIEx/Bac-1-UBIAD1 point mutants. 1 μL of template (50 ng/mL) was mixed with 25 μL of 2 × PCR buffer for KOD FX (TOYOBO Company Ltd., Osaka, Japan), 10 μL of 2 mM dNTP (dATP, dGTP, dTTP, dCTP) mixed solution, 1 μL of 1 U/μL KOD FX, 1.5 μL *Sal*I (Takara Bio Inc., Otsu, Japan) and Kozak sequence-anchored sense primer (*Sal*I_hUBIAD1_atg: AAAGTCGACCATGGCGGCCTCTCAGGTCCTG), 1.5 μL *Not*I-anchored antisense primer (*Not*I_hUBIAD1_taa: AAGCGGCCGCTTAAATTTTGGGCAGACTGCC) and 10 μL sterilized ultra-pure water. PCR amplification was performed using the T100 thermal cycler system (BioRad). After digestion with *Sal*I and *Not*I, the insert DNA fragment and pEBMulti-neo vector were obtained by electrophoresis (Cosmo Bio Company Ltd., Tokyo, Japan). The ligation reaction was performed with 4 μL insert DNA fragment and 2 μL pEBMulti-neo vector using Ligation High ver.2 (TOYOBO) to generate pEBMulti-UBIAD1 and pEBMulti-UBIAD1 point mutants.

### MG63 cell transfection

Cells were cultured to 1 × 10^5^ cells/10 mL/10 cm dish for 24 h without antibiotics in an incubator at 37°C and 5% CO_2_. Cell confluency reached 40–60% before nucleofection. 10 μg of pEBMulti-UBIAD1 or pEBMulti-UBIAD1 point mutants was used for stable cell transfection overnight. The entire cell transfection procedure was performed according to the Lipofectamine 2000 manual (Invitrogen). On the next day, the medium was replaced with DMEM medium containing 10% FCS and 10 μL of G418 0.5 mg/mL. When cell confluency reached 80–90%, the cells were passed once. One-time passed cells were used for stable-cell-transfected pEBMulti-UBIAD1 (wild type) or pEBMulti-UBIAD1 point mutants.

### Cellular cholesterol measurement for the N-ethyl-N-(2-hydroxy-3-sulfopropyl)-3,5-dimethoxyaniline sodium salt assay

Stable cells transfected with pEBMulti-UBIAD1 or pEBMulti-UBIAD1 point mutants were cultured for 48 h in six-well plates in an incubator at 37°C, 5% CO_2_. Cells were washed with PBS and each well was treated with 100 μL lipid lysis buffer (50 mM Tris-HCl, pH 7.4; 1 mM EDTA; 150 mM NaCl, 10 mM NaF, 10 mM Na_2_P_2_O_7_, plus 1% Triton X-100, 100 μM phenylarsine oxide and protease inhibitor mixture). The obtained sample’s cellular cholesterol content was determined using a total cholesterol measurement kit (Wako).

### Real-time PCR

Total RNA of MG63 cells was isolated using Isogen (Nippon Gene, Tokyo, Japan) according to the manufacturer’s protocol. First-strand cDNA synthesis was performed using ReverTra Ace (TOYOBO). cDNAs were mixed with THUNDERBIRD qPCR Mix (TOYOBO) and amplified using the CFX96 real-time PCR system (BioRad).

### Localization of human UBIAD1 and UBIAD1 mutant

A human UBIAD1 cDNA fragment was inserted into the recombination region of the pIEx-UBIAD1 or the pIEx-UBIAD1 mutant vector to create pEBMulti-UBIAD1 or the pEBMulti-UBIAD1 mutant (Wako). MG63 cells were transfected with 10 μg of pEBMulti-UBIAD1 using the Lipofectamine 2000 reagent and cloned in a selective medium containing 500 μg ml^−1^ G418 (Nacalai). Cloned pEBMulti-UBIAD1 or pEBMulti-UBIAD1 mutant cells were harvested on a glass-bottom dish, cultured for 2 days. As a control, we used the empty pEBMulti vector. UBIAD1 mutant cells were stained with the UBIAD1 antibody, and Golgi bodies were stained with GOLGA5 (Sigma-Aldrich, St. Louis, MO, USA). The nuclei were stained with DAPI. Cells were fixed with 10% formaldehyde solution, viewed under an LSM 700 microscope and photographed (Carl Zeiss, Thornwood, NY, USA).

### Measurements of CoQ9, CoQ10, ^13^C_6_-CoQ9, ^13^C_6_-CoQ10 and Solanesyl- *p*-hydroxybenzoic acid (SPHB)

Cell lysates (1 mL) were transferred into a brown-glass tube with a Teflon-lined screw cap. Next, we added 1 mL of ethanol containing MK-4-^18^O as the internal standard, 1 mL of ethanol and 3 mL of hexane. After thorough mixing on a voltex mixer for 5 min, the mixture was centrifuged at 1,500 × g for 5 min at 4°C and the upper layer was transferred to a small brown-glass tube and evaporated to dryness under reduced pressure. The residue was dissolved in 2 mL of hexane and evaporated under reduced pressure. This residue was dissolved in 100 μL of methanol. An aliquot of this solution was analysed by APCI3000 LC-MS/MS (Applied Biosystems, Foster City, CA, USA). HPLC analyses were performed on a Shimadzu HPLC system (Shimadzu Co. Ltd., Kyoto, Japan) consisting of a binary pump (LC-10AD liquid chromatography), an automatic solvent degasser (DGU-14A degasser) and an auto-sampler (SIL-10AD autoinjector). Separations were performed using a reversed-phase C18 column (COSMOSIL 5C_18_ AR-II, 5 μm; 4.6 mm inner diameter × 150 mm, Nacalai) with a solvent system consisting of isocratic solvent A, containing methanol:isopropanol (3:1, v/v) and was delivered at 1.0 mL/min. The column was maintained at 35°C using a column oven (CTO-10AC column oven). All MS data were collected in positive ion mode with atmospheric pressure chemical ionisation (APCI). The following settings were used: corona discharge needle voltage of 5.5 kV, vaporizer temperature of 400°C, sheath gas (high-purity nitrogen) pressure of 50 p.s.i. and transfer capillary temperature of 220°C. The electron multiplier voltage was set at 850 eV. Identification and quantification were based on MS/MS using the multiple reactions monitoring mode. The range for the parent scan was 400–900 atomic mass units. The multiple reactions monitoring transitions (precursor ion and product ion, m/z) and retention time (minutes) for each analyte were as follows: MK-4-^18^O precursor ion: 449.3, product ion: 191.2, retention time: 3.6; CoQ9 precursor ion: 796.5, product ion: 197.1, retention time: 8.7, CoQ10 precursor ion: 864.6, product ion: 197.0, retention time: 11.5; ^13^C_6_-CoQ9 precursor ion: 802.5, product ion: 203.1, retention time: 8.6; ^13^C_6_-CoQ10 precursor ion: 870.6, product ion: 203.0, retention time: 11.4; SPHB precursor ion: 751.6, product ion: 151.0, retention time: 6.7; and ^13^C_6_-SPHB precursor ion: 757.6, product ion: 157.0, retention time: 6.7 [16, 36–38]. Calibration using internal standardisation was performed by linear regression using five different concentrations: 100, 200, 400, 800 and 1,600 ng/mL.

### Conversion of MD-d_8_ to MK-4-d_7_ and ^13^C_6_-PHB to ^13^C_6_-CoQ10 in MG63 cells

MG63 cells were cultured in six-well tissue culture plates (2 × 10^5^ cells/well) for 24 h and treated with culture medium containing MD-d_8_ (10^−6^ M) and ^13^C_6_-PHB for 24 h. Transfected MG63 cells were trypsinised, washed with cold PBS(−) twice and stored at −30°C. After heating to room temperature, cells were lysed in 1 mL of PBS(−). Cell lysates (20 μL) were analysed for protein concentrations. PK-^18^O and MK-4-^18^O were added as internal standards to the cell lysates in brown screw-capped tubes. Measurements for MK-4-d_7_, MK-4-d_7_ epoxide, CoQ10 and ^13^C_6_-CoQ10 in cells were performed using the method described above.

### Preparation of mitochondrial fraction from Sf9 cells expressing COQ2

The cDNA for the COQ2 coding region was cloned into pIEx/Bac-1 (pIEx/Bac-1-COQ2). Recombinant baculovirus expressing COQ2 was generated using the BacMagic system (Merck) according to the manufacturer’s protocol. To achieve the COQ2 expression, cell cultures (2 × 10^6^ cells/ mL) were infected of recombinant baculovirus, followed by a 72 h expression period. Cells were collected and washed three times with cold PBS(−) and then stored at −30°C. For the experiments, cells were thawed to room temperature and resuspended in 10× cell volume of sonication buffer containing 100 mM Tris-HCl (pH 7.4), 500 mM NaCl and 1% Proteinase inhibitor cocktail. The cell suspension was sonicated for 5 min and centrifuged at 3,000 × g for 10 min to remove unbroken cells and nuclei. The supernatant was centrifuged at 8,000 × g for 10 min. The mitochondria pellet was resuspended in ice-cold buffer A containing 100 mM Tris-HCl (pH 7.4) and protease inhibitor mix. The mitochondrial suspension was homogenized and stored at −80°C.

### Conversion of MD-d_8_ to MK-4-d_7_ and ^13^C_6_-PHB to ^13^C_6_-SPHB or ^13^C_6_-CoQ9 in the mitochondrial fraction derived from Sf9 cells expressing COQ2

Reactions were performed in 1 mL of 100 mM Tris-HCl (pH 7.4), 1 mM DTT, 100 nM GGPP, 100 nM MD-*d*_8_, 100 nM ^13^C_6_-PHB, 100 nM solanesol and 10 μg protein of the Sf9-COQ2 mitochondrial fraction. Reaction mixtures were incubated at 37°C for 3 h, and the reactions were stopped by the addition of 1 mL ethanol. Metabolites were extracted with 3 mL hexane and 1 mL ethanol containing the internal standard. The metabolites were analysed by LC-APCI-MS/MS as described above.

### Statistical analysis

Data are expressed as mean ± SEM. Differences between the mean values were analysed using the unpaired Student’s *t*-test or Dunnett’s test: *P < 0.05; **P < 0.01; ***P < 0.001 and Tukey–Kramer's honestly significant difference (HSD) test: Values not sharing a common letter in each group are significantly different (p <0.05).

## Results

### Enzyme reaction conditions with UBIAD1-expressing Sf9 cell microsomes

**Fig 1:**
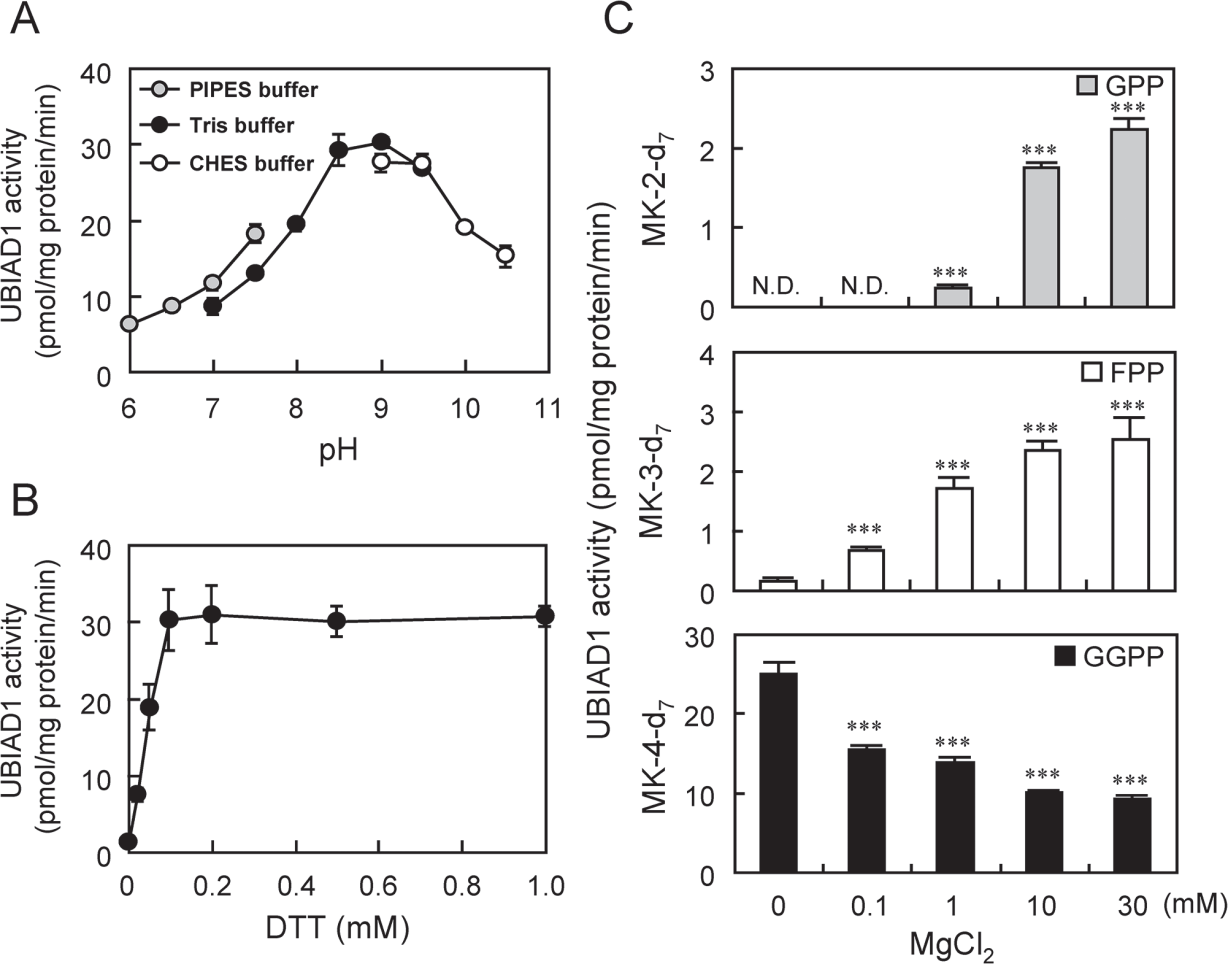
Human UBIAD1 Prenyltransferase Activity. (A) Effect of DTT on prenyltransferase activity of human UbiA prenyltransferase domain-containing protein 1 (UBIAD1). (B) pH dependence of prenyltransferase activity of human UBIAD1. Grey circles indicate the PIPES buffer (pH 6.0–7.5; 100 mM), black circles the Tris buffer (pH 7.0–9.5; 100 mM) and white circles indicate the CHES buffer (pH 9.0–10.5; 100 mM). (C) Magnesium dependence of prenyltransferase activities of human UBIAD1. Three kinds of prenyldiphosphates, GPP (grey bar), farnesyl pyrophosphate (FPP) (white bar) and GGPP (black bar), were used as substrates. Significantly different from cells treated with 0-mM MgCl_2_; ***P < 0.001, Dunnett’s test. N.D.: not detected. (D) Effect of various divalent metal ions on prenyltransferase activities of human UBIAD1. Significantly different from non-treated cells; ***P < 0.001, Dunnett’s test. N.D.: not detected.

UBIAD1 is a membrane protein present in the endoplasmic reticulum [14, 15] and Golgi complex [15, 16]. To define the enzymological properties of UBIAD1, including optimum pH, reductant requirement, substrate recognition characteristics and metal requirement, we used endoplasmic reticulum-rich microsomal fractions from UBIAD1-expressing Sf9 cells as an enzyme source. First, we determined the optimum pH for the MK-4 synthetic activity of UBIAD1 (Fig 1A). Within the range of pH 6.0–7.5 (PIPES buffer) and pH 7.0–9.0 (Tris buffer), MK-4 synthesis increased with the elevation of pH. However, MK-4 synthesis decreased in the pH range of 9.0 to 10.5 (CHES buffer). Therefore, we considered the optimum pH for the MK-4 synthetic activity of UBIAD1 to be between pH 8.5 and 9.0 and used pH 8.5 Tris buffer afterwards in this study. Next, we evaluated DTT requirements for MK-4 synthetic activity of UBIAD1 (Fig 1B). No MK-4 synthetic activity of UBIAD1 was detected in the absence of DTT. Adding DTT stimulated the MK-4 synthetic activity of UBIDA1 and maximum activity was detected with 0.1 mM DTT. Because higher DTT levels did not further increase the enzymatic activity, we used 1 mM in the study. Although the microsomal fraction from UBIAD1-expressing Sf9 cells synthesizes MK-4-d_7_ from MD-d_8_ using GGPP as an isoprenyl side-chain substrate, other isoprenyl side-chains are produced by the mevalonate pathway, including GPP and FPP. It is assumed that UBIAD1 recognizes GPP or FPP as side-chain substrates to synthesize MK-2 or MK-3, respectively. We examined the production of MK-2-d_7_ and MK-3-d_7_ from MD-d_8_ by UBIAD1 with GPP or with FPP as a side-chain substrate. We also examined the substrate selectivity and Mg^2+^ requirement of UBIAD1 (Fig 1C). Using GGPP as the side-chain substrate, the MK-4 synthetic activity was decreased when Mg^2+^ levels increased. On the other hand, with GPP as the side-chain substrate, MK-2-d_7_ production increased dose dependently, with MgCl_2_ levels ranging from 1 mM to 30 mM. With FPP as the side-chain substrate, MK-3-d_7_ production increased in a dose-dependent manner as MgCl_2_ levels ranged from 0.1 mM to 30 mM.

### Effects of mevalonate pathway inhibitors on MK-4 synthesis

**Fig 2:**
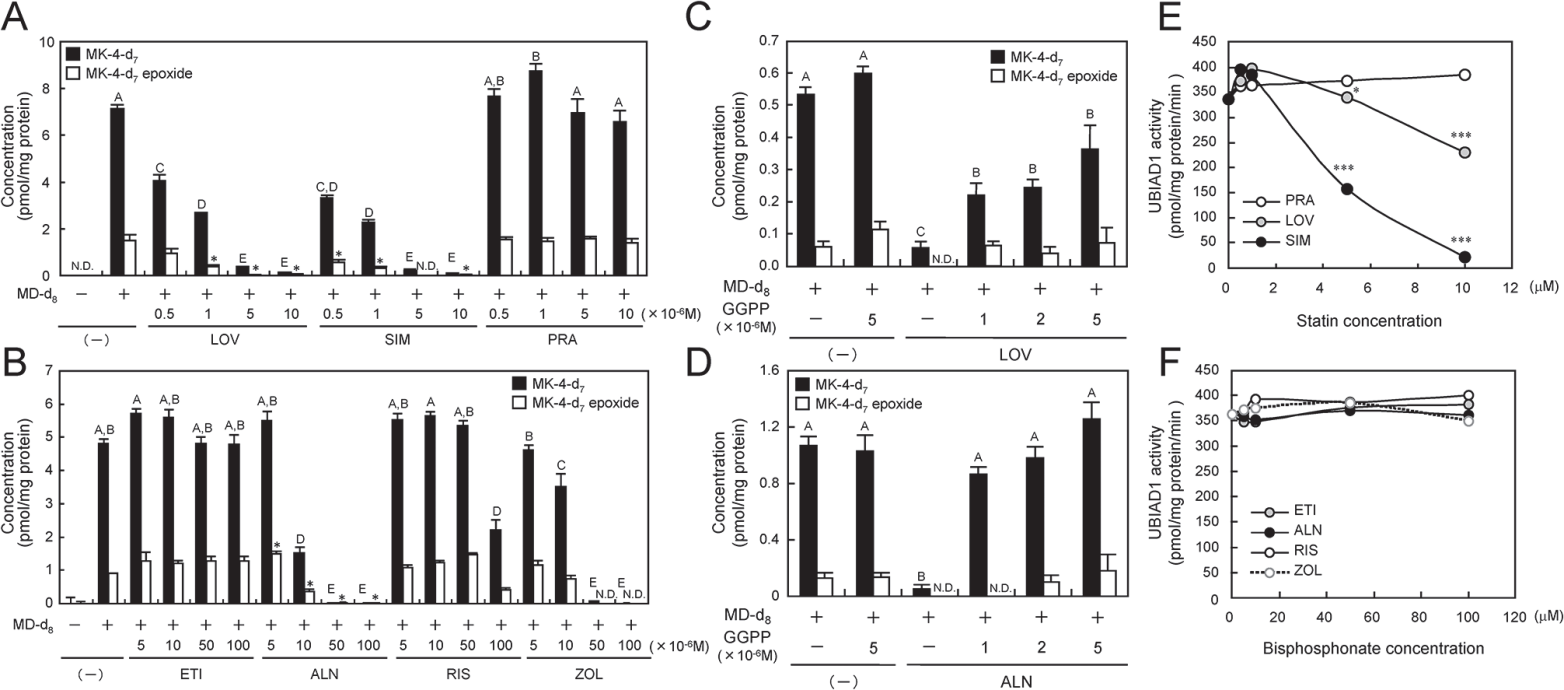
Influence of Statins and Bisphosphonates on Human UbiA Prenyltransferase Domain-containing Protein 1 Activity. (A) Effect of statins on MK-4 conversion activity in MG63 cells. (B) Effect of bisphosphonates on MK-4 conversion activity in MG63 cells. Values not sharing a common letter in each group are significantly different (Tukey–Kramer HSD-test): P < 0.05. Significantly different from MD-d_8_-treated cells; *P < 0.05, Dunnett’s test. N.D.: not detected. (C) Geranylgeranyl diphosphate (GGPP) dose-dependent MK-4 conversion activity of UBIAD1 in statin-treated MG63 cells. (D) GGPP dose-dependent MK-4 conversion activity of UBIAD1 in bisphosphonate-treated MG63 cells. Values not sharing a common letter in each group are significantly different (Tukey–Kramer HSD-test): P < 0.05. N.D.: not detected. (E) Statin dependence of prenyltransferase activities of UBIAD1 in Sf9-UBIAD1 microsomes. Significantly different from non-treated cells; *P < 0.05, ***P < 0.001, Dunnett’s test. (F) Bisphosphonates dependence of prenyltransferase activities of UBIAD1 in Sf9-UBIAD1 microsomes.

When UBIAD1 synthesises MK-4 from MD, it uses GGPP as a source of geranylgeranyl side-chain. GGPP is a product of the mevalonate pathway. The mevalonate pathway can be inhibited by statins and bisphosphonates, leading to a decrease of GGPP production. Statins decrease GGPP levels by 3-hydroxy-3-methylglutaryl-coenzyme A reductase (HMGCR) inhibition and bisphosphonates by FPP synthesis inhibition. Therefore, we examined the effects of both the mevalonate pathway inhibitors on MK-4 synthesis using a human osteoblast-like cell line, MG63. MG63 cells are responsive to vitamin K and UBIAD1-expressing Sf9 cell microsomes. First, we investigated the effects of statins (LOV, SIM or PRA) on MK-4-d_7_ and MK-4-d_7_ epo productions from MD-d_8_ in MG63 cells. By adding lipophilic statins, LOV or SIM, MK-4-d_7_ and MK-4-d_7_ epo production levels were significantly decreased in a dose-dependent manner. No change in MK-4-d_7_ and MK-4-d_7_ epo production levels was observed when adding the aqueous statin PRA (Fig 2A). Next, we evaluated the effects of bisphosphonates (ETI, ALN, RIS or ZOL) on MK-4-d_7_ and MK-4-d_7_ epo productions from MD-d_8_ in MG63 cells. By adding nitrogen-containing bisphosphonates, ALN or ZOL, MK-4-d_7_ and MK-4-d_7_ epo production levels were significantly decreased in a dose-dependent manner, and a significant decrease in MK-4-d_7_ production level was observed when adding 10^−4^ M of RIS. On the other hand, no change in the MK-4-d_7_ production level was observed when adding the non-nitrogen-containing bisphosphonate ETI (Fig 2B). We examined the effects of GGPP addition on MK-4-d_7_ synthesis in MG63 cells treated with the mevalonate inhibitors LOV and ALN. As a result, MK-4-d_7_ production levels were significantly decreased in the presence of either mevalonate inhibitor, but simultaneous addition of GGPP significantly enhanced MK-4-d_7_ production levels dose dependently (Fig 2C and 2D). However, in the LOV-treated MG63 cells, MK-4 production levels were not completely recovered even with excessive amounts of GGPP, suggesting that LOV could directly inhibit MK-4 synthetase UBIAD1. Thus, we examined the effects of statins with UBIAD1-expressing Sf9 cell microsomes. As a result, the lipophilic statins LOV and SIM significantly inhibited MK-4 production in a dose-dependent manner (Fig 2E), whereas the aqueous statin PRA induced no significant change in MK-4 production. In addition, bisphosphonates were found to inhibit the conversion into MK-4-d_7_ (Fig 2F).

### MK-4 synthetic activity in UBIAD1 mutant-expressing Sf9 cell microsomes

**Fig 3:**
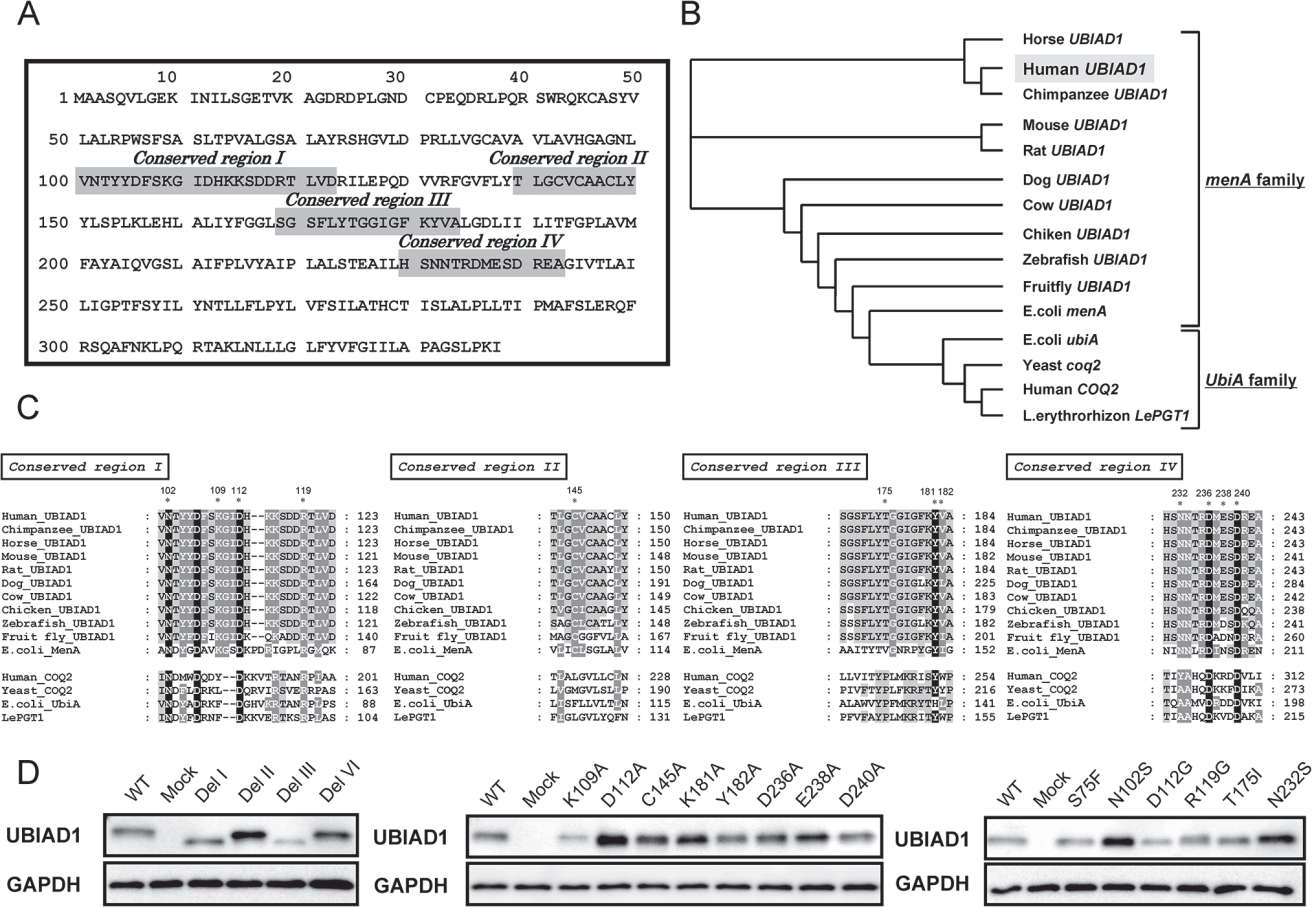
Representative Features of the Human UBIAD1 Protein and Multiple Alignment of Regions Conserved among Prenylation Enzyme Family Members involved in Menaquinone and Coenzyme Q Synthesis. (A) Highly concerved region of human UbiA prenyltransferase domain-containing protein 1 (UBIAD1). Gray box indicates the highly concerved region. (B) Phylogenetic analysis of the UbiA prenyltransferase family. (C) Amino acid residues corresponding to Regions I, II, III and IV. (D) Immunoblotting of human UBIAD1 expressed in Sf9 cells microsomal proteins. The UBIAD1 band size is 36.8 kDa and the GAPDH band size is 36.5 kDa. WT:wild type.

Recently, Cheng *et al*. and Huang *et al*. have defined the crystal structure of UbiA derived from archaebacteria, followed by a detailed structural analysis of the UbiA family [34, 35]. However, because human UBIAD1 does not belong to the UbiA family but to the menA family based on the phylogenetic tree (Fig 3A and 3B), no detailed information about the prenylation domain or substrate recognition site in the aminoacid sequence is available. Structural features of UBIAD1 could be identified by analysis of the UBIAD1 aminoacid sequence. Furthermore, because UBIAD1 is the gene responsible for SCD, the point mutation of UBIAD1 was reported in patients with SCD. Therefore, introducing point mutations in UBIAD1 followed by the analyses of enzymatic activities changes should help to identify key activity sites of UBIAD1. Thus, we analysed the homology between human UBIAD1 and highly homologous prenylation enzymes to identify the highly conserved domains and aminoacids of UBIAD1.

### Homology analysis of UBIAD1 and expression of the UBIAD1 mutant protein

We analysed the homology of the aminoacid sequences across human, mammalian and non-mammalian UBIAD1s; *E*. *coli* menA; and the COQ2 homolog using ClustalW and generated a phylogenetic tree using Tree View (Fig 3A and 3B). Homology analysis with ClustalW followed by detection of highly homologous aminoacids using GeneDoc detected four domains sharing high aminoacid sequence homology among the UBIAD1s, *E*. *coli* menA, and COQ2. Several aminoacids were found to be highly conserved in these domains (Fig 3C). Based on these findings, we constructed UBIAD1 mutants deficient in these domains, produced mutants in which we substituted alanine for the most conserved aminoacids in each conserved domain and also substituted the mutant variant reported in patients with SCD by Orr *et al* [20]. We examined UBIAD1 protein expressions in the microsomes from Sf9 cells overexpressing the UBIAD1 conserved domain deletion mutants and UBIAD1 point mutants. Sf9 cell microsomes infected with the control virus is referred to as ‘mock’, with the UBIAD1 virus referred to as ‘wild type’ and the UBIAD1 mutant as ‘name of the mutation site’. Del I, Del II, Del III and Del IV mutants were found to express a UBIAD1 protein of lower molecular weight compared with the ‘wild type’. The different alanine substitution mutants and SCD mutants had the same molecular weight of 36.8 kDa as the wild type (Fig 3D).

### Prenylation activity in the microsomes from Sf9 cells overexpressing the UBIAD1 mutant

**Fig 4:**
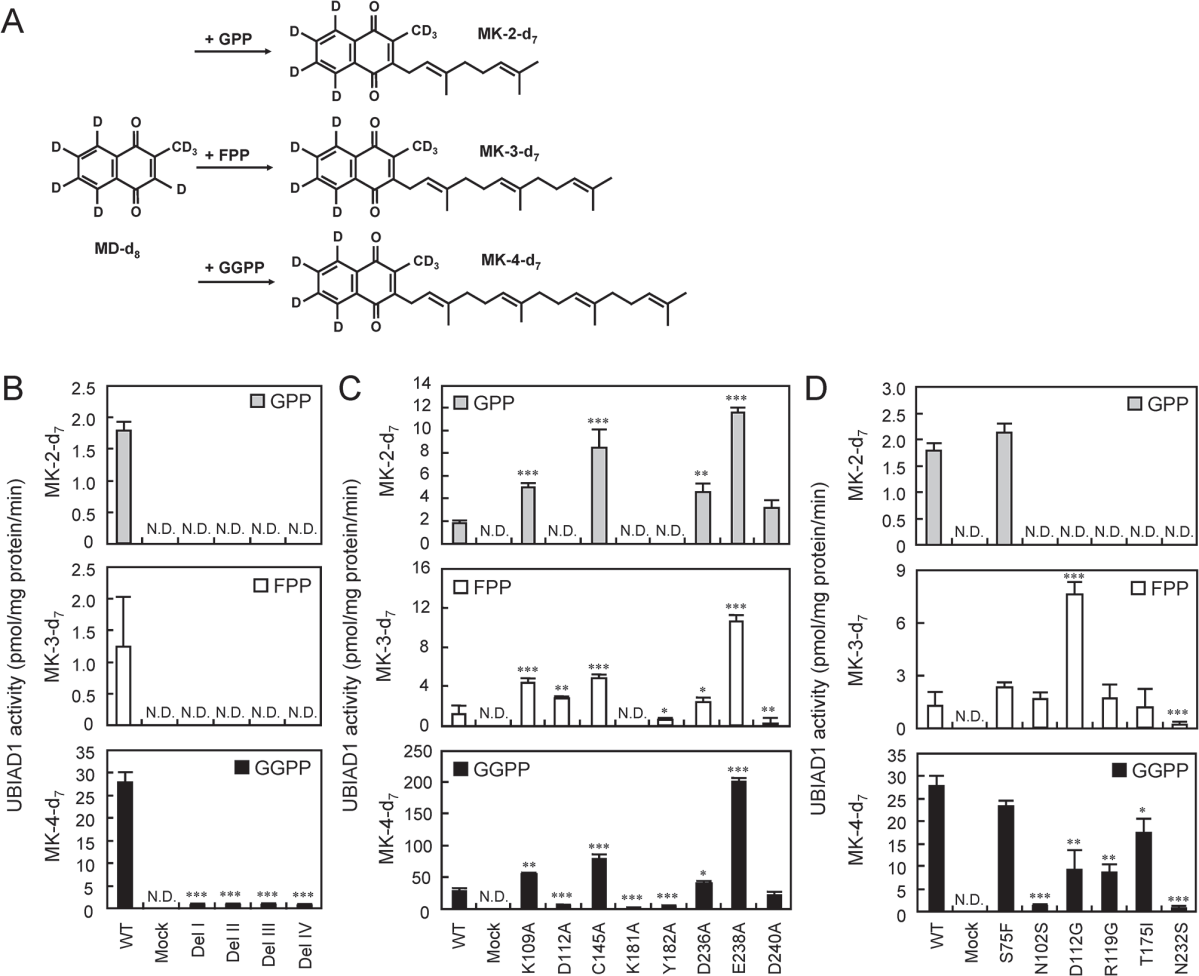
MK-n Synthesis Activity of Human UBIAD1 Mutants of Conserved Aminoacids. (A) Schematic representation the prenylation mechanisms of MD (MD-d_8_) conversion to menaquinones (MK-n-d_7_). (B) MK-n synthetic activity of human UbiA prenyltransferase domain-containing protein 1 (UBIAD1) deletion mutants. Significantly different from wild type (WT) UBIAD1; ***P < 0.001, Dunnett’s test. N.D.: not detected. (C) MK-n synthetic activity of human UBIAD1 point mutants by alanine scanning. Significantly different from wild type UBIAD1; *P < 0.05, **P < 0.01, ***P < 0.001, Dunnett’s test. N.D.: not detected. (D) MK-n synthetic activity of human UBIAD1 point mutants caused by Schnyder corneal dystrophy. Significantly different from wild type UBIAD1; *P < 0.05, **P < 0.01, ***P < 0.001, Dunnett’s test. N.D.: not detected.

First, we examined enzyme activities in the microsomes from Sf9 cells overexpressing the UBIAD1 conserved domain deletion mutants. Wild type UBIAD1 converts MD-d_8_ to MK-2-d_7_, MK-3-d_7_ and MK-4-d_7_ with GPP, FPP and GGPP as a side-chain substrate, respectively, with the expected activity (Fig 4A). However, the activity of UBIAD1 conserved domain deletion mutant conversion of MD-d_8_ to MK-4-d_7_ with substrate GGPP was minimal (Fig 4B). Next, we examined enzyme activities in the microsomes from Sf9 cells overexpressing the alanine substitution UBIAD1 point mutants (Fig 4C). With GPP as the side-chain substrate, normal activity was detected during the conversion of MD-d_8_ to MK-2-d_7_ in wild type UBIAD1, but it completely diminished in D112A, K181A or Y182A mutants. However, in K109A, C145A, D236A, E238A or D240A mutants, similar or higher activity than that wild type was detected. With FPP as the side-chain substrate, normal activity was detected during the conversion of MD-d_8_ to MK-3-d_7_ in wild type UBIAD1. The activity was markedly decreased in K181A, Y182A or D240A mutants, but it significantly increased in K109A, D112A, C145A, D236A or E238A mutants. With GGPP as the side-chain substrate, normal activity was detected during the conversion of MD-d_8_ to MK-4-d_7_ in wild type UBIAD1, but it markedly decreased in D112A, K181A or Y182A mutants. However, the activity was significantly increased in K109A, C145A, D236A or E238A mutants. In addition, we examined enzyme activities of microsomes from Sf9 cells overexpressing the SCD-UBIAD1 point mutants (Fig 4D). With GPP as a side-chain substrate, normal activity in converting MD-d_8_ to MK-2-d_7_ was observed in wild type UBIAD1, but markedly decreased in N102S, D112G, R119G, T175I or N232S. With GPP as the side-chain substrate, conversion of MD-d_8_ to MK-2-d_7_ was normal in wild type UBIAD1 but markedly decreased in N102S, D112G, R119G, T175I, and N232S mutants. With FPP as the side-chain substrate, conversion of MD-d_8_ to MK-3-d_7_ was similarly normal in wild type UBIAD1. The activity was markedly decreased in the UBIAD1 N232S point mutant but significantly increased in D112G. With GGPP as the side-chain substrate, conversion of MD-d_8_ to MK-4-d_7_ was normal in normal UBIAD1 but markedly decreased in N102S, D112G, R119G, T175I, and N232S mutants. S75F is a point mutant unrelated to SCD.

### Kinetics analysis in the microsomes from Sf9 cells overexpressing the UBIAD1 point mutant

We analysed the enzyme reaction kinetics in K109A, D112A, D112G, C145A and E238A mutants (Table 1). For the point mutants K109A, C145A and E238A in which enzyme activity was increased with GPP, FPP or GGPP substrates, the *K*m value for MD-d_8_ was increased and the affinity was decreased. Because *V*max was markedly increased, enzymatic activity was considered to be increased. *K*m for GPP was increased and the affinity was decreased in K109A, C145A and E238A mutants. However, enzyme activity was considered to be increased according to the increased *V*max. *K*m for FPP and GGPP in K109A was similar to that in the wild type and was slightly decreased in C145A and E238A. Based on these results, the affinity of these mutants for FPP and GGPP was considered to be similar to or higher than that for the wild type. In addition, the activity for synthesising MK-3 and MK-4 was considered to be greatly increased because *V*max was also increased. For the mutants D112A and D112G in which enzyme activity was increased only with FPP as the side-chain, the *K*m value for MD-d_8_ was increased and *V*max was markedly decreased, suggesting that enzyme activity was significantly decreased. Moreover, the affinity for GPP was completely diminished. *K*m for FPP and GGPP was decreased and the affinity was slightly increased.

**Table 1:** Kinetic Parameters of Wild Type and Human UBIAD1 Mutants for MK-n Synthesis Activity.

Substrate	UBIAD1	*V*max	*K*m	*V*max/*K*m
	mutation	(pmol/min/mg protein)	(nM)	(×10^−3^)
K_3_-d_8_	WT	34.2 ± 2.7	28.3 ± 4.1	1.24
	K109A	75.3 ± 6.2	41.2 ± 3.2	1.87
	D112A	9.5 ± 0.6	46.2 ± 8.0	0.22
	D112G	31.7 ± 3.9	117.7 ± 10.5	0.27
	C145A	119.7 ± 3.4	54.5 ± 8.1	2.33
	E238A	245.9 ± 16.0	41.0 ± 3.2	6.03
GPP	WT	3.6 ± 0.3	187.6 ± 25.7	0.02
	K109A	6.7 ± 0.6	274.8 ± 39.5	0.02
	D112A	N.D.	N.D.	N.D.
	D112G	N.D.	N.D.	N.D.
	C145A	21.9 ± 2.3	242.2 ± 14.0	0.09
	E238A	22.0 ± 0.4	271.2 ± 47.2	0.09
FPP	WT	3.5 ± 0.1	46.6 ± 2.0	0.08
	K109A	6.4 ± 0.1	44.7 ± 1.0	0.14
	D112A	3.4 ± 0.4	26.0 ± 1.3	0.13
	D112G	4.6 ± 0.3	42.4 ± 3.2	0.11
	C145A	12.1 ± 0.7	36.4 ± 2.5	0.34
	E238A	17.0 ± 2.9	32.4 ± 1.5	0.41
GGPP	WT	37.2 ± 0.5	175.3 ± 15.8	0.22
	K109A	75.6 ± 8.1	180.8 ± 38.3	0.44
	D112A	15.8 ± 0.4	58.8 ± 7.3	0.28
	D112G	38.1 ± 3.8	81.3 ± 3.4	0.47
	C145A	111.6 ± 5.3	143.2 ± 6.1	0.79
	E238A	223.1 ± 9.5	129.2 ± 4.8	1.73

N.D.: not detected. WT: wild type

### Relationship between MK-4 synthetic activity of UBIAD1 and cholesterol synthesis

**Fig 5:**
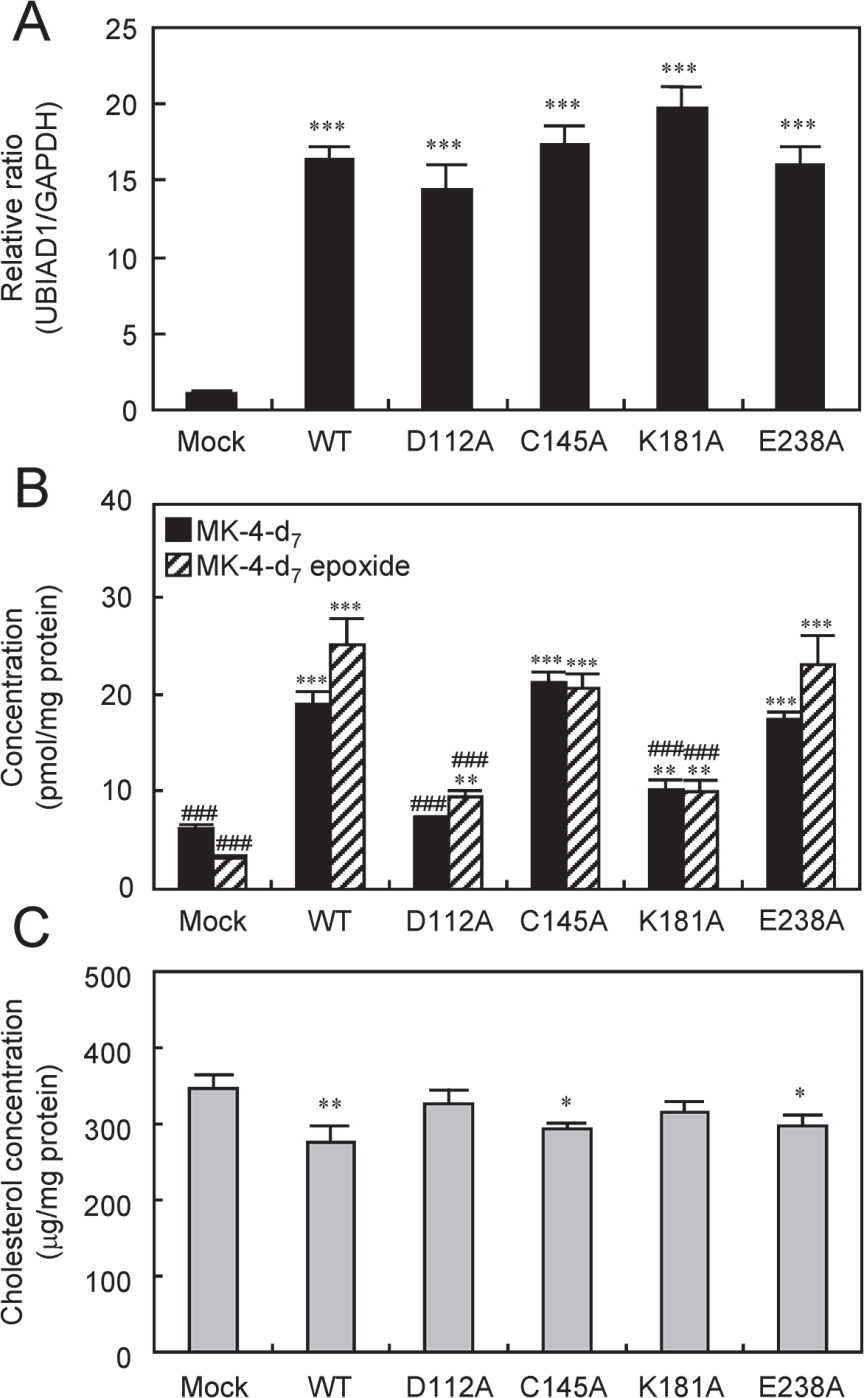
MK-4 Synthetic Activity and Cholesterol Biosynthesis Activities of Human UBIAD1 Point Mutants by Alanine Scanning in MG63 Cells. (A) mRNA expression in human UbiA prenyltransferase domain-containing protein 1 (UBIAD1) point mutants by alanine scanning. Significantly different from control; ***P < 0.001, Dunnett’s test. WT:wild type. (B) MK-4 synthetic activity of human UBIAD1 point mutants by alanine scanning. Significantly different from control; **P < 0.01, ***P < 0.001, Dunnett’s test. Significantly different from wild type; ^###^P < 0.001, Student’s *t*-test. (C) Cholesterol biosynthetic activity of human UBIAD1 point mutants by alanine scanning. Significantly different from the control; *P < 0.05, **P < 0.01, Student’s t test.

SCD, which causes deposition of cholesterol in the cornea, results from a point mutation of UBIAD1 [17, 39]. Therefore, we evaluated the relationship between MK-4 synthetic activity of UBIAD1 and cholesterol synthesis to reveal the relation between the pathology of SCD and MK-4 synthesis. Point mutants in each of the four UBIAD1 conserved domains were stably transfected into MG63 cells, which is responsive to vitamin K. The MG63 point mutant and wild type UBIAD1-expressing MG63 cells showed similar UBIAD1 mRNA levels (Fig 5A). Measurement of the conversion level of MD-d_8_ to MK-4-d_7_ in these cells using LC-APCI-MS/MS showed that the amount of MK-4-d_7_ and MK-4-d_7_ epoxide production were significantly higher in the wild type UBIAD1 and C145A and E238A mutants than in the control. However, D112A and K181A produced the same amount of MK-4-d_7_ and MK-4-d_7_ epoxide as the control. Therefore, the tendency observed in these results and in those of the microsomes from Sf9 cells is the same (Fig 5B). In addition, the measurement of intercellular cholesterol levels revealed that they were significantly reduced in the wild type, C145A and E238A compared with the control, but these levels were not reduced in D112A and K181A (Fig 5C).

### Subcellular localization of UBIAD1

**Fig 6:**
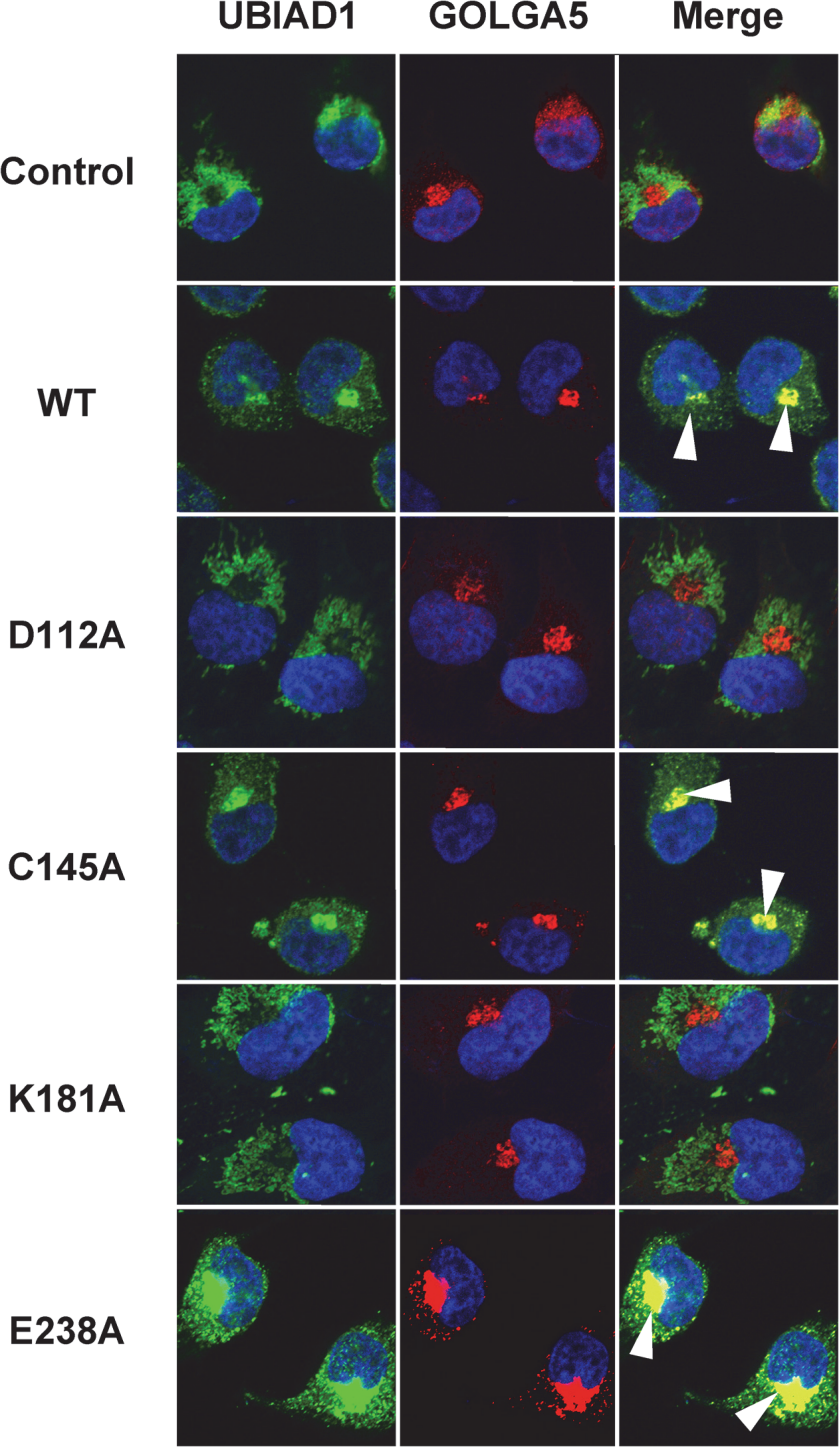
Subcellular Localization of Human UBIAD1 in MG63 cells. MG63 cells were transfected with pEBMulti-UBIAD1 mutant. Cells were then stained with UBIAD1 antibody (Green) and the Golgi apparatus was stained with GOLGA5 (Red). As a control, we used the empty vector pEBMulti (Control). Cells were viewed under a LSM 700 microscope and photographed. All experiments were repeated at least three times. White arrows indicate areas with both green and red staining. WT: wild type.

Nakagawa *et al*. have previously demonstrated by immunostaining that GFP-fused UBIAD1 was localized to the endoplasmic reticulum of human osteoblast-like cells MG63 [14]. However, by immunostaining, Nickerson *et al*. reported that UBIAD1 was localized in the mitochondria of parenchymatous cells of the human cornea, and Vos *et al*. found mitochondrial localization in drosophila-derived S2 cells [10, 17]. Furthermore, using immunostaining and cell fraction methods with human endothelium cells, Mugoni *et al*. found that UBIAD1 was localized to the Golgi body but not to the mitochondria [16]. In a recent study using HEK293 human embryonic kidney cells and T24 bladder carcinoma cells, Wang *et al*. revealed that UBIAD1 had a novel Golgi localization signal RPWS sequence, and it was localized to the endoplasmic reticulum and Golgi body [15]. Taken together, the localization site of UBIAD1 might be dependent on the cell type. Therefore, we analysed the subcellular localization of UBIAD1 using aminoacid mutants in the four conserved domains, which influenced MK-4 synthetic activity and cholesterol synthetic activity. This revealed that D112A and K181A did not localize to the Golgi body. In contrast, wild type UBIAD1, C145A, and E238A were highly localized to the Golgi body (Fig 6).

## Discussion

UBIAD1 is a membrane protein localized to the endoplasmic reticulum [14, 15] and Golgi complex [15,16]. In general, purification of membrane proteins is difficult, especially when the structure of the enzyme’s activite site must be kept intact [40, 41]. We attempted to purify UBIAD1 by extracting the endoplasmic reticulum-constituent-rich microsomal fraction from UBIAD1-expressing Sf9 cells, but we failed to purify it such that its MK-4 synthetic activity is retained. Mugoni *et al*. previously reported that UBIAD1 was a CoQ synthetase in genetically modified zebrafish *ubiad1* and human endothelial cells [16]. Thus, we analysed the synthesis rate of human CoQ10 using MG63 cells in which the *UBIAD1* gene was knocked down or overexpressed. In order to eliminate the influence of endogenous CoQ10, ^13^C_6_-CoQ10 levels were analysed after the addition of ^13^C_6_-4HB; however, ^13^C_6_-CoQ10 levels were unchanged regardless of the amount of the *UBIAD1* gene (S1 Fig). Using the mitochondrial fraction from Sf9 cells overexpressing the *COQ2* gene, we evaluated the amount of ^13^C_6_-CoQ9 synthesised, which is highly synthesised in insect cells, after adding ^13^C_6_-4HB. These data indicated that although a large amount of ^13^C_6_-CoQ9 was detected in the *COQ2* gene-expression mitochondrial fraction, ^13^C_6_-CoQ9 was barely detected in the *UBIAD1* gene-expressing microsomes. Nakagawa *et al*. reported that in *ubiad1* gene deletion mouse ES cells, no vitamin K synthesis was detected but the amount of CoQ9 synthesised was unchanged [36]. Then, we evaluated the enzymological properties of UBIAD1 proteins based on the vitamin K synthetic activity using microsomal fractions from UBIAD1-expressing Sf9 cells as an enzyme source.

First, we found that the optimum pH for the MK-4 synthetic activity of UBIAD1 ranges between pH 8.5 and 9.0. Further, we demonstrated that MK-4 synthetic activity was the highest in the presence of reductant DTT and in the absence of metals. The isoelectric point was calculated based on the UBIAD1 aminoacid sequence as 8.4. Because the optimum pH for an enzyme is usually close to its isoelectric point, the optimum pH for UBIAD1 is expected to be basic. We confirmed experimentally that UBIAD1 activity was the highest under basic conditions. With regard to the reductant DTT, it is assumed to reduce MD to the hydroquinone form. When synthesising MK-4 chemically, MD is unable to bind GGPP in a non-reduced state. Thus, reduction of MD is required for prenylation by UBIAD1. In general, enzymes with prenylation activities, including ubiA, ubiquinone synthetase and naringenin 8-dimethylallyltransferase and prenylation enzymes in plants, require Mg^2+^ [42]. However, for MK-4 synthesis with GGPP as the side-chain substrate, large amounts of Mg^2+^ inhibit the activity. Our prenylation assay had one limitation: the presence of small amounts of Mg^2+^ derived from the Sf9 microsome. Therefore, we cannot suggest that this assay would work in the absence of Mg^2+^. GPP and FPP have shorter isoprene units than GGPP, and using either of these as the side-chain substrate produced MK-2 and MK-3 in the presence of Mg^2+^. The selectivity of isoprenyl compounds recognized by UBIAD1 may be explained by interactions between isoprenyl compounds and amino acids constituting the side-chain recognition site of UBIAD1 in the absence or presence of Mg^2+^ [43]. Other prenylation enzymes recognizing GPP and FPP are known to utilize Mg^2+^ as a linker to stimulate the binding between GPP or FPP and the substrate compound. Similarly, UBIAD1 might require Mg^2+^ for reaction with GPP or FPP. However, because GGPP has more repeated isoprene units and a larger hydrophobic region than GPP or FPP, it may directly interact with amino acids in the side-chain recognition site of UBIAD1. Alternatively, the shorter distance between the terminal phosphate group of GGPP and MD may allow UBIAD1 to prenylate MD in the presence of lower concentrations of Mg^2+^. Heide reports that many membrane-bound prenyltransferases attach isoprenoid moieties derived from short-chain allylic isoprenyl diphosphates, such as dimethylallyl diphosphate (C5), GPP (C10) or FPP (C15), to an acceptor molecule in the presence of Mg^2+^. However, soluble prenyltransferases (such as Ras farnesyltransferase) attach GGPP (C20) to an acceptor protein in the absence of Mg^2+^ [42]. Although we do not know the amount of Mg^2+^ required for prenylation, our results suggest that higher concentrations of Mg^2+^ may exert an inhibitory effect on UBIAD1 activity when GGPP is used as the side-chain substrate.

Because GGPP produced by the mevalonate pathway was used as a side-chain source in MK-4 synthesis of UBIAD1, we examined the role of this pathway in MG63 cells using mevalonate pathway inhibitors. We demonstrated that the amount of MK-4 synthesised was markedly decreased in cells treated with lipophilic statins (HMG-CoA reductase inhibitors) and nitrogen-containing bisphosphonates (FFP synthetase inhibitors). These effects were mostly rescued by adding GGPP. However, in the LOV-treated MG63 cells, MK-4 production levels were not completely recovered even with excessive amounts of GGPP, suggesting that LOV might directly inhibit MK-4 synthetase UBIAD1. Therefore, we investigated the inhibitory potential of statins and bisphosphonates against UBIAD1. We found that UBIAD1 activity was significantly decreased with LOV and SIM, suggesting a direct inhibition of the UBIAD1 enzyme by these lipophilic statins. Using the yeast two-hybrid system and immunoprecipitation, Nickerson *et al*. have reported that HMGCR binds to UBIAD1 at the protein level [39]. Based on this report, it is considered that the binding of statins to HMGCR causes a change in the coupling between HMGCR and UBIAD1, resulting in reduced MK-4 synthetic activity. However, this is unlikely because UBIAD1 activity was unaffected by aqueous statins, which have a similar affinity for HMGCR. Analysis of the LOV-coupled HMGCR crystal structure has demonstrated that LOV strongly binds to 12 aminoacids of HMGCR with hydrogen bonds and van der Waals force, thus inhibiting HMGCR enzymatic activity [44]. The aminoacid sequence homology of UBIAD1 and HMGCR is only 12%; however, UBIAD1 contains the same three aminoacids required for LOV binding as HMGCR. We propose that lipophilic statins may inhibit UBIAD1 activity by directly binding specific aminoacids of UBIAD1.

After defining the basic characteristics of the UBIAD1 protein and the enzymatic reaction conditions for MK-4 synthesis from MD, we identified key domains and aminoacids responsible for the MK-4 synthesis in UBIAD1. The resulting enzyme activities were completely eliminated in all the UBIAD1 conserved domain deletion mutants, thus indicating that each of these four domains is essential for enzymatic activity. Furthermore, our aminoacid sequence homology analysis of menA and ubiA families led to the identification of key aminoacids that are strongly conserved across species and are responsible for the MK-4 synthesis in UBIAD1. We analysed enzyme activities by generating alanine mutants for K109 and D112 present in the UBIAD1 conserved domain I, C145 in the UBIAD1 conserved domain II, K181 and Y182 in the UBIAD1 conserved domain III, and for D236, E238 and D240 in the UBIAD1 conserved domain IV. We also analysed enzyme activities of the UBIAD1 point mutants reported in N102S, D112G, R119G, T175I, N232S and patients with SCD, as well as a point mutant unrelated to SCD, S75F, as a control [35].

In the UBIAD1 conserved domain I, the mutant K109A resulted from substitution of the 109^th^ basic aminoacid with polarity, lysine, to the low molecular hydrophobic amino acid, alanine. The increased hydrophobicity significantly enhanced the affinity for the hydrophobic sites of FPP and GGPP, thus increasing enzyme activities. On the other hand, D112A only showed MK-3 synthetic activity with FPP as the side-chain substrate. We believe that the change of the large sterically-hindered-CH_2_COOH of aspartic acid to-CH_3_ of alanine reduced the steric interference, allowing the farnesyl side-chain to bind freely with MD. In addition, the *K*m value of D112A was smaller than that of UBIAD1 with FPP as the side-chain substrate, indicating a better substrate recognition for FPP. Although the SCD mutation S75F was found to have the same prenylation activity as UBIAD1, enzyme activities of N102S and R119G were significantly reduced with both isoprenyl side-chains and only D112G showed increased activity with FPP as the side-chain source. The *K*m value of D112G was smaller than that of UBIAD1, indicating high substrate recognition of FPP. Thus, we considered that the bonding with FPP was enhanced and activities were retained. Altogether, MK-4 synthetic activity is significantly decreased in the point mutants causing SCD. Recently, Huang *et al*. reported that based on the crystal structure of AfUbiA, N102 in the UBIAD1 conserved domain I was a binding site to Mg^2+^/isoprenyl side-chain and that D112 and R119 were key aminoacids associated with structural changes by binding to substrates. These results show that the UBIAD1 conserved domain I is the substrate recognition site for vitamin K synthesis.

Weiss *et al*. reported that the UBIAD1 conserved domain II containing a cysteine at position 145 had a sequence similar to the redox site CxxC motif in vitamin K reductase VKORC1 [45]. The activities in C145A were increased with both isoprenyl side-chains and this might be explained by the fact that the non-polar alanine stabilized its charge.

For K181A and Y182A in the UBIAD1 domain III, enzyme activities were significantly reduced. This domain is located almost at the centre of the UBIAD1 protein and is considered to be an important hinge region of the secondary structure. In G177E, we substituted the peripheral aminoacid glycine to glutamic acid; however, no target protein could be obtained (date not shown). Interestingly, besides the full length UBIAD1 consisting of 338 aminoacids, a second UBIAD1 isoform was reported in which the termination codon at position 180 results from a frameshift mutation of aminoacid 177 [39]. Previous studies have reported that K181 is a binding site for the Mg^2+^/isoprenyl side-chain and G177 is located in the vicinity of the enzyme’s active site. In addition, for the SCD mutation T175I, enzyme activity was significantly reduced with both isoprenyl side-chains. T175 is located in a region containing many polar groups and is considered to be a catalytic site of the enzyme. Therefore, the UBIAD1 conserved domain III is an essential region of the UBIAD1 enzyme active centre.

Melzer *et al*. have reported that the UBIAD1 conserved domain IV is located next to the prenylation domain in the conserved domain I and is an important structure for the normal function of the enzyme [46]. Mutating D236, E238 and D240 in domain IV to alanine increased the activities. Mutating polar hydrophilic aminoacid to non-polar hydrophobic alanine is suggested to enhance protein stability. This is evident from the diminution of most activities in two point mutants K109A and T175I (S2 Fig). In addition, for N232S reported in patients with SCD, enzyme activity was significantly reduced with both isoprenyl side-chains. The UBIAD1 conserved domain IV is rich in aspartic acid and has a structure similar to that of the FARM motif (DDxxxxD) in the FPP synthesis enzyme. Because D236 and D240 are binding sites to the Mg^2+^/ isoprenyl side-chain, the UBIAD1 conserved domain IV is considered to be an isoprenyl side-chain binding site (S3 Fig).

Because SCD caused by mutations in the *UBIAD1* gene is a disorder of cholesterol metabolism, MK-4 synthetic activity is assumed to influence cholesterol metabolism [17, 20, 39]. We used MG63 cells, under physiological conditions close to those in humans, responsive to UBIAD1 expression and having a normal cholesterol metabolic system. We measured the cholesterol levels in D112, C145, K181 and E238 alanine mutants. Although in D112A and C145A, MK-4 synthetic activity was reduced, cholesterol was increased. This suggested that MK-4 synthetic activities of UBIAD1 are linked to intercellular cholesterol synthetic ability. Cholesterol is synthesized via GPP, FPP and squalene by the mevalonate pathway [47, 48]. MK-4 is simultaneously synthesized by UBIAD1 with GGPP, which is produced from FPP by GGPP synthetase, as the side-chain source. This indicates that cholesterol and MK-4 share FPP as a source factor produced by the mevalonate pathway. Based on this, it is likely that when MK-4 synthetic activity was increased with enhanced UBIAD1 expression, large amounts of GGPP were consumed as a side-chain source of MK-4, thus using the GGPP precursor FPP for GGPP synthesis and consequently decreasing cholesterol synthesis. In contrast, when UBIAD1 was knocked down by RNAi in the adipocyte precursor cell line (3T3-L1), cholesterol levels were increased as confirmed by oil red O staining (date not shown). Thus, in UBIAD1 point mutants, a decrease in MK-4 synthetic activity allows FPP to be used for cholesterol synthesis more than for GGPP synthesis, elevating the cholesterol level. In addition, Nicekerson *et al*. have pointed out that in the study with B cells derived from patients with SCD in which corneal cholesterol deposition occurs, reduction in MK-4 synthetic activity is one of the factors responsible for intercellular cholesterol accumulation. Our study confirms the role of UBIAD1 function in cholesterol synthesis.

Several reports on subcellular localization of UBIAD1 have been published and suggested that subcellular localization varies depending on cell types [10, 14–17]. Therefore, we examined the subcellular localization of UBIAD1 point mutants in each of the four conserved domains, which we have shown to have an influence on MK-4 synthetic activity and cholesterol synthesis. We found that D112A and K181A were not localized to the Golgi body as in the control. However, wild type UBIAD1, C145A, and E238A were highly localized to the Golgi body (Fig 6). Mugoni *et al*. and Wang *et al*. also found that UBIAD1 was localized to the Golgi body. It is unclear why little localization to the Golgi body was observed for D112A and K181A [15, 16]. Wang *et al*. reported that UBIAD1 had the Golgi body localization signal RPWS and was synthesized in the endoplasmic reticulum, followed by transfer to the Golgi body via coat protein complex II. One hypothesis may be that coat protein complex II formation is inhibited in D112A and K181A mutants. Subcellular mislocalization of UBIAD1 affects both MK-4 and cholesterol synthesis.

With regard to enzymological properties and structural features of UBIAD1, it is very likely that MK-4 synthetic activity greatly impacts cholesterol synthesis and subcellular localization. Although the crystal structures of the UbiA homolog from *A*. *pernix* and AfUbiA have been identified, the three-dimensional structure of human UBIAD1 remains to be determined [34, 35]. X-ray crystal structure analysis with purified the UBIAD1 protein retaining its MK-4 synthetic activity may enable to uncover further mechanisms underlying UBIAD1. In addition, the precise molecular mechanisms by which UBIAD1 is involved in cholesterol synthesis should be analysed. We believe that further investigation of the physiology of UBIAD1 may reveal the relationship of UBIAD1 with cholesterol synthesis and SCD pathology.

## Supporting Information

S1 Fig Ubiquinone Biosynthesis by COQ2 or UBIAD1. (A) Schematic representation of CoQ9/CoQ10 biosynthetic mechanisms of COQ2 or UBIAD1. (B) CoQ10 biosynthetic activity in MG63 cells. Conversion of MD-d_8_ to MK-4-d_7_ and ^13^C_6_-PHB to ^13^C_6_-CoQ10 in MG63 cells transfected with *UBIAD1* siRNA or *UBIAD1* expression vector. (C) CoQ9 biosynthetic activity in cellular organelle fraction derived from Sf9 cells expressing *COQ2* or *UBIAD1*.
(TIF)

S2 Fig MK-n Synthesis Activity of Human UBIAD1 Double Point Mutants. (A) Western blot analysis of human UBIAD1 double point mutants expressed in Sf9 cells. The UBIAD1 band size is 36.8 kDa and the GAPDH band size is 36.5 kDa. WT:wild type. (B) MK-n synthetic activity of human UBIAD1 double point mutants. Three kinds of prenyldiphosphates GPP (grey bar), farnesyl pyrophosphate (FPP) (white bar) and GGPP (black bar) were used as substrates.
(TIF)

S3 Fig Schematic Representation of the Putative Secondary Structure of UBIAD1. Locations of this study’s mutations in a proposed two-dimensional model of UBIAD1 in a lipid bilayer. Grey cycle: deletion mutants, red cycle: point mutants by alanine scanning, blue cycle: point mutants by Schnyder corneal dystrophy (SCD) mutations and yellow cycle: D112 by alanine scanning and SCD mutations. The conserved domain I is a substrate recognition site where a structural change was induced by substrate binding. In particular, D112 was predicted to be a key substrate recognition site for the synthesis of MK-3 from farnesyl pyrophosphate (FPP). The conserved domain II is a redox domain site containing a CxxC motif. The conserved domain III is a hinge region and is important as a catalytic site for the UBIAD1 enzyme. The conserved domain IV is a binding site for Mg^2+^/isoprenyl side-chain.
(TIF)

S4 Fig Reaction Kinetics of MK-n Synthesis Activity of Wild Type and Human UBIAD1 Mutants. The upper number above each bar is *Vmax* (pmol/min/mg protein). The lower number above each bar is *Km* (nM). *Vmax*/*Km* (× 10^−3^) values of WT and UBIAD1 mutants were determined from Lineweaver–Burk plots. N.D.: not detected. WT: wild type (A) K_3_-d_8_, (B) GPP, (C) FPP, (D) GGPP.
(TIF)

S1 Table Oligonucleotides used in the Present Study. (XLS)
