# Supplementary figures and images for: Functional Characterization of the Vitamin K2 Biosynthetic Enzyme UBIAD1
Source: PLoS One. 2015 Apr 15;10(4):e0125737. doi: 10.1371/journal.pone.0125737 (PMC4398444; doi:10.1371/journal.pone.0125737)

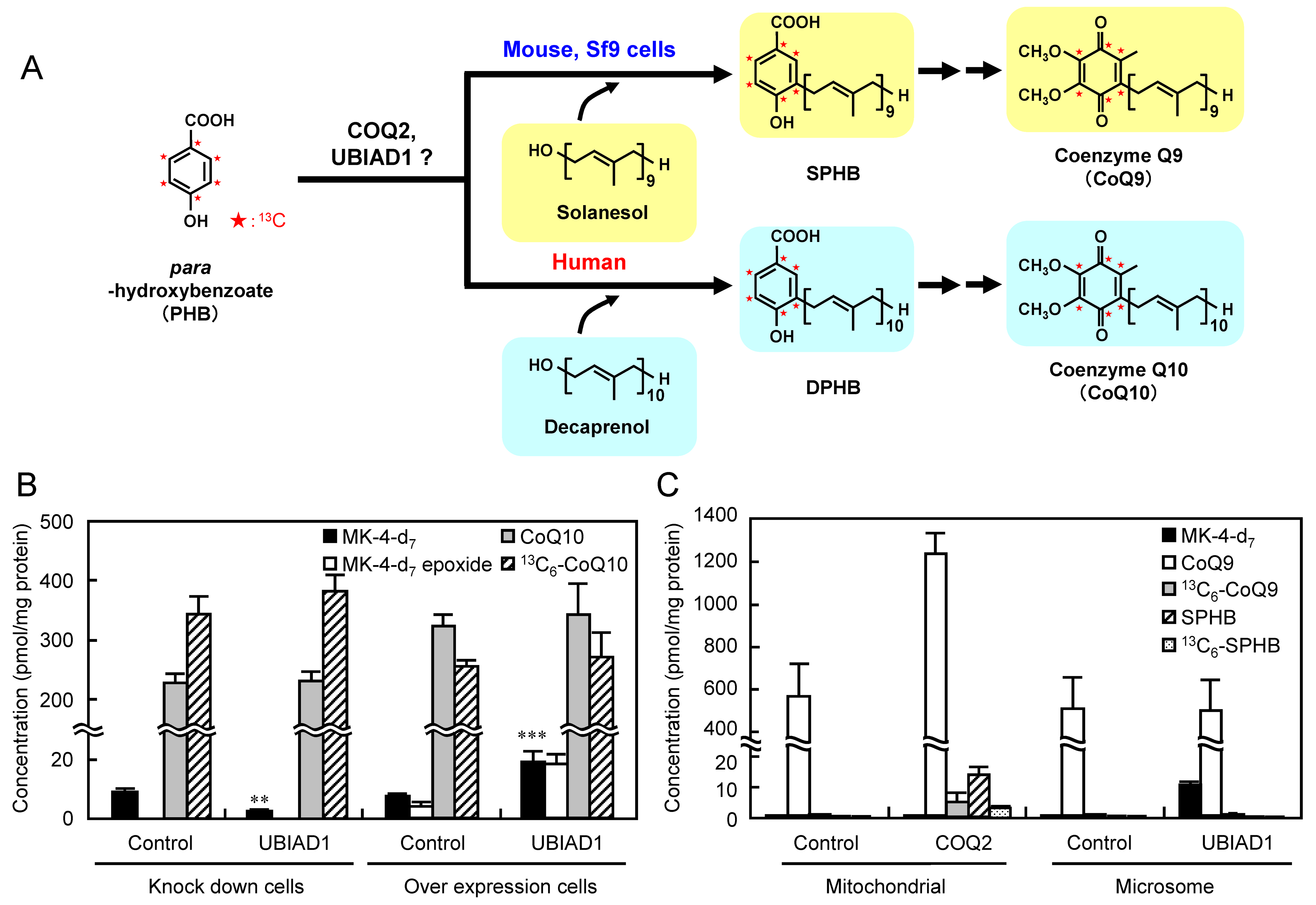

Supplement: S1 Fig — (A) Schematic representation of CoQ9/CoQ10 biosynthetic mechanisms of COQ2 or UBIAD1. (B) CoQ10 biosynthetic activity in MG63 cells. Conversion of MD-d8 to MK-4-d7 and 13C6-PHB to 13C6-CoQ10 in MG63 cells transfected with UBIAD1 siRNA or UBIAD1 expression vector. (C) CoQ9 biosynthetic activity in cellular organelle fraction derived from Sf9 cells expressing COQ2 or UBIAD1. (TIF) [file pone.0125737.s001.tif]

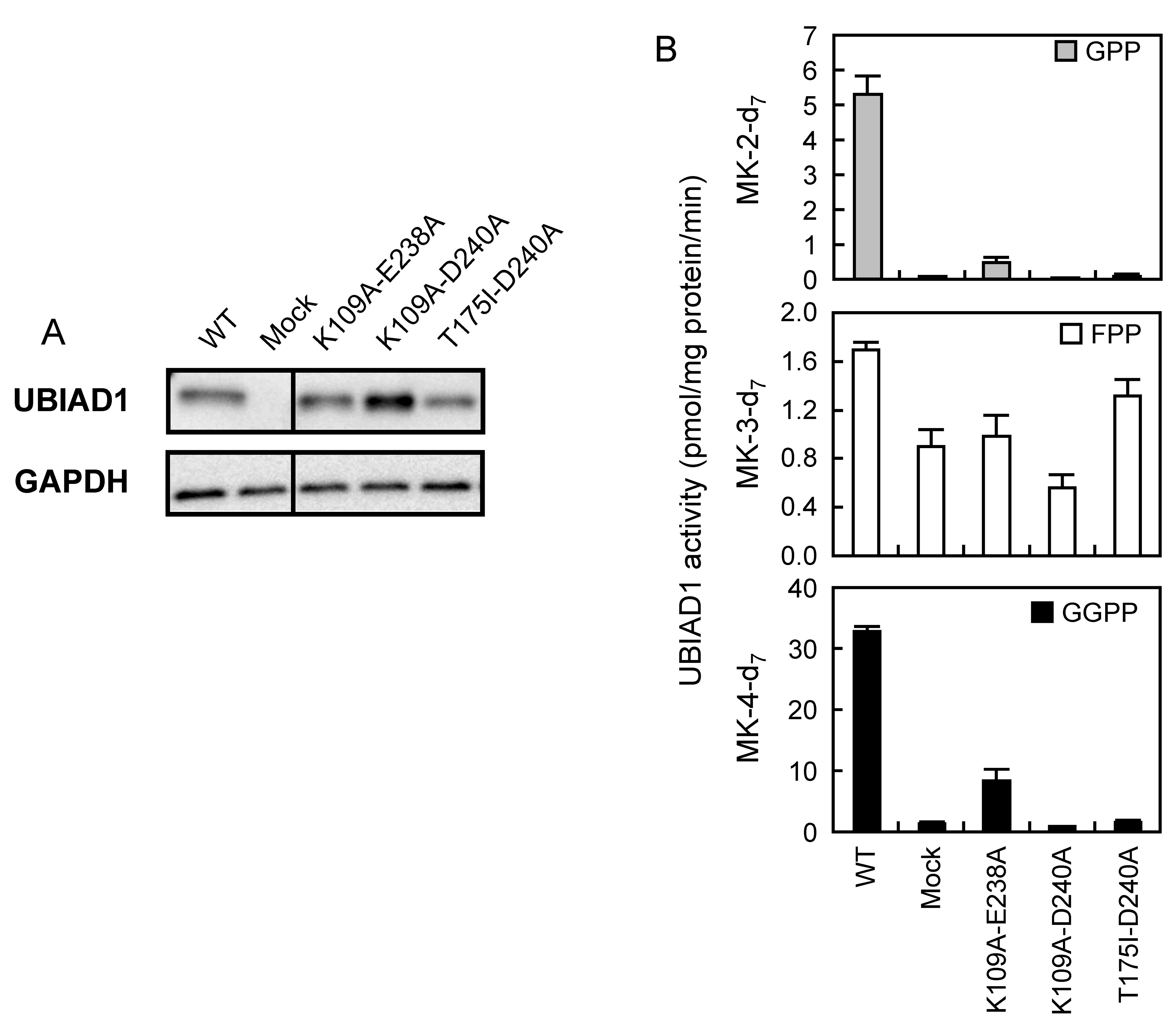

Supplement: S2 Fig — (A) Western blot analysis of human UBIAD1 double point mutants expressed in Sf9 cells. The UBIAD1 band size is 36.8 kDa and the GAPDH band size is 36.5 kDa. WT:wild type. (B) MK-n synthetic activity of human UBIAD1 double point mutants. Three kinds of prenyldiphosphates GPP (grey bar), farnesyl pyrophosphate (FPP) (white bar) and GGPP (black bar) were used as substrates. (TIF) [file pone.0125737.s002.tif]

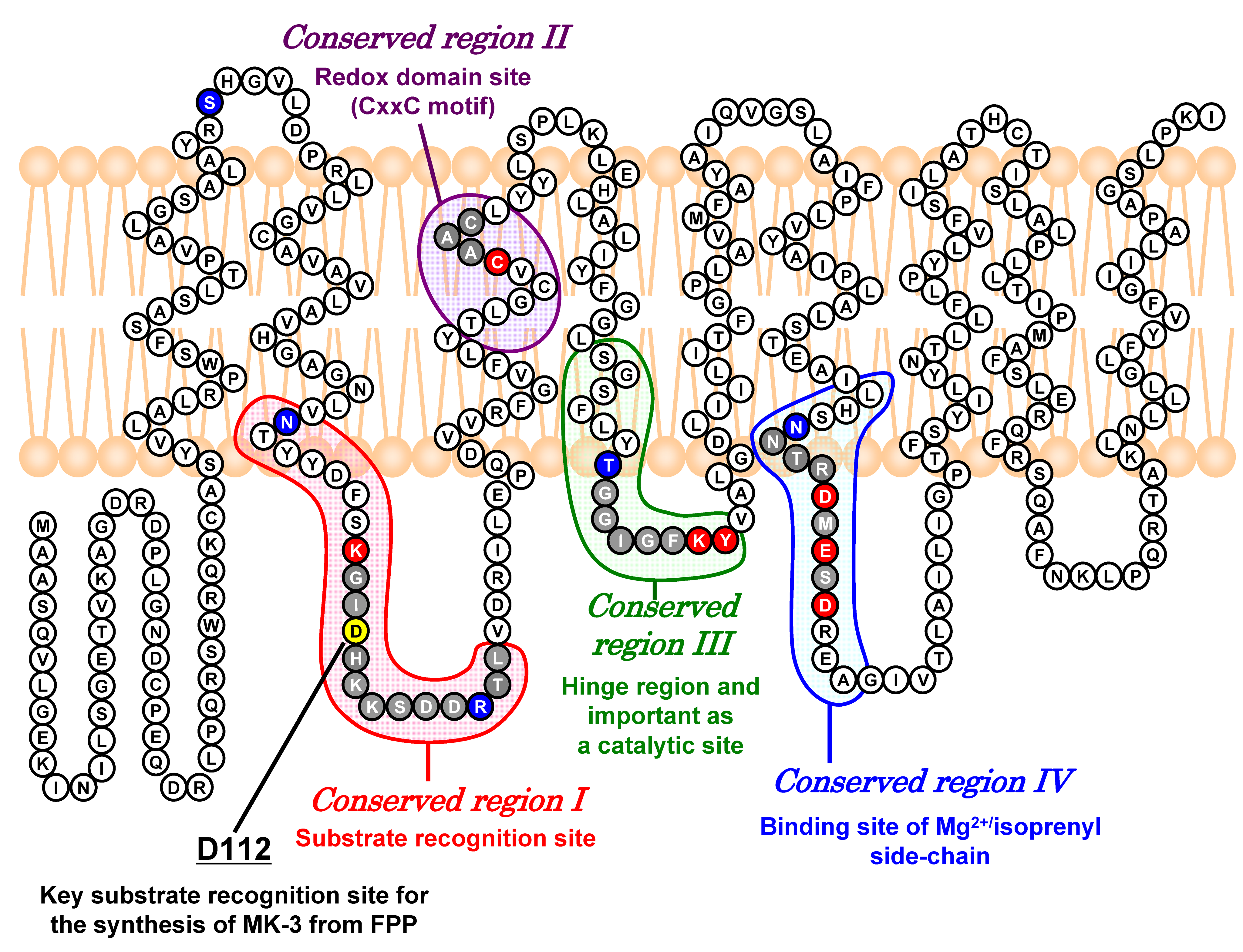

Supplement: S3 Fig — Locations of this study’s mutations in a proposed two-dimensional model of UBIAD1 in a lipid bilayer. Grey cycle: deletion mutants, red cycle: point mutants by alanine scanning, blue cycle: point mutants by Schnyder corneal dystrophy (SCD) mutations and yellow cycle: D112 by alanine scanning and SCD mutations. The conserved domain I is a substrate recognition site where a structural change was induced by substrate binding. In particular, D112 was predicted to be a key substrate recognition site for the synthesis of MK-3 from farnesyl pyrophosphate (FPP). The conserved domain II is a redox domain site containing a CxxC motif. The conserved domain III is a hinge region and is important as a catalytic site for the UBIAD1 enzyme. The conserved domain IV is a binding site for Mg2+/isoprenyl side-chain. (TIF) [file pone.0125737.s003.tif]

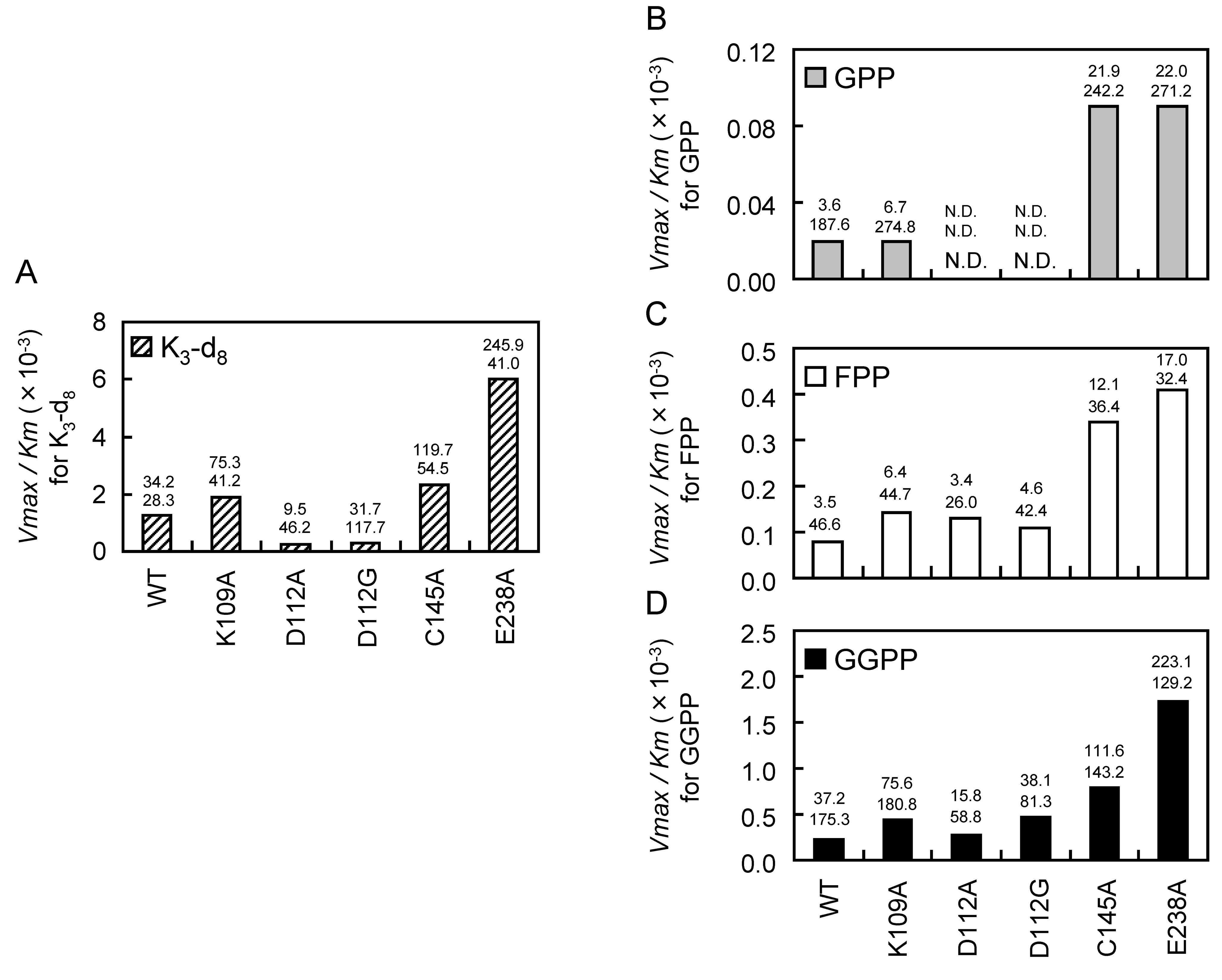

Supplement: S4 Fig — The upper number above each bar is Vmax (pmol/min/mg protein). The lower number above each bar is Km (nM). Vmax/Km (× 10−3) values of WT and UBIAD1 mutants were determined from Lineweaver–Burk plots. N.D.: not detected. WT: wild type (A) K3-d8, (B) GPP, (C) FPP, (D) GGPP. (TIF) [file pone.0125737.s004.tif]
